# Disability in childhood and the equity of health services: a cross-sectional comparison of mass drug administration strategies for soil-transmitted helminths in southern Malawi

**DOI:** 10.1136/bmjopen-2023-083321

**Published:** 2024-09-05

**Authors:** Stefan Witek-McManus, James Simwanza, Rejoice Msiska, Hastings Mangawah, William Oswald, Joseph Timothy, Sean Galagan, Emily Pearman, Mariyam Shaikh, Hugo Legge, Judd Walson, Lazarus Juziwelo, Calum Davey, Rachel Pullan, Robin L Bailey, Khumbo Kalua, Hannah Kuper

**Affiliations:** 1 Department of Disease Control, London School of Hygiene and Tropical Medicine, London, UK; 2 Blantyre Institute for Community Outreach, Blantyre, Malawi; 3 Department of Global Health, University of Washington, Seattle, Washington, USA; 4 National Schistosomiasis and STH Control Programme, Community Health Sciences Unit, Ministry of Health & Population, Lilongwe, Malawi; 5 Department of Population Health, London School of Hygiene and Tropical Medicine, London, UK; 6 Department of Clinical Research, London School of Hygiene and Tropical Medicine, London, UK; 7 Kamuzu University of Health Sciences, Blantyre, Malawi

**Keywords:** Neglected Diseases, Mass Drug Administration, Schools, Disabled Persons

## Abstract

**ABSTRACT:**

**Background:**

School-based approaches are an efficient mechanism for the delivery of basic health services, but may result in the exclusion of children with disabilities if they are less likely to participate in schooling. Community-based ‘door to door’ approaches may provide a more equitable strategy to ensure that children with disabilities are reached, but disability is rarely assessed rigorously in the evaluation of health interventions.

**Objectives:**

To describe the prevalence and factors associated with disability among children aged 5–17 years and to assess the relative effectiveness of routine school-based deworming (SBD) compared with a novel intervention of community-based deworming (CBD) in treating children with disabilities for soil-transmitted helminths.

**Setting:**

DeWorm3 Malawi Site (DMS), Mangochi district, Malawi.

**Participants:**

All 44 574 children aged 5–17 years residing within the DMS.

**Primary and secondary outcome measures:**

Disability was defined as a functional limitation in one or more domains of the Washington Group/UNICEF Child Functioning Module administered as part of a community-based census. Treatment of all children during SBD and CBD was independently observed and recorded. For both intervention types, we performed bivariate analyses (z-score) of the absolute proportion of children with and without disabilities treated (absolute differences (ADs) in receipt of treatment), and logistic regression to examine whether disability status was associated with the likelihood of treatment (relative differences in receipt of treatment).

**Results:**

The overall prevalence of disability was 3.3% (n=1467), and the most common domains of disability were hearing, remembering and communication. Boys were consistently more likely to have a disability compared with girls at all age groups, and disability was strongly associated with lower school attendance and worse levels of education. There was no significant difference in the proportion of children with disabilities treated during SBD when assessed by direct observation (−1% AD, p=0.41) or likelihood of treatment (adjusted risk ratio (aRR)=1.07, 95% CI 0.89 to 1.28). Treatment of all children during CBD was substantially higher than SBD, but again showed no significant difference in the proportions treated (−0.5% AD, p=0.59) or likelihood of treatment (aRR=1.04, 95% CI 0.99 to 1.10).

**Conclusion:**

SBD does not appear to exclude children with disabilities, but the effect of consistently lower levels of educational participation of children with disabilities should be actively considered in the design and monitoring of school health interventions.

**Trial registration number:**

NCT03014167.

**STRENGTHS AND LIMITATIONS OF THIS STUDY**

- This study was conducted with a large, community-representative population and included rigorous assessment of disability using a standardised and validated tool (Washington Group/UNICEF Child Functioning Module).
- This study assessed receipt of deworming treatment using independent direct observation, rather than less reliable measures of treatment such as self-report or proxy response which are often used to assess mass drug administration coverage.
- Linking children observed during school-based deworming to their census record was challenging with many treated children not identified, although this misclassification is likely to be non-differential with respect to disability status.
- Community-based deworming was implemented in the context of a cluster-randomised trial, and as such our results may not be generalisable to routine community-based interventions.

## Introduction

 There are an estimated 240 million children with disabilities globally, equal to one in every 10 children.^1^ Evidence consistently demonstrates that children with disabilities experience substantial disparities across a broad range of health^2^,^4^ and education^5 6^ outcomes compared with children without disabilities. Having previously been neglected within the global development agenda, addressing disability is becoming recognised as critical to achieving the objective of Universal health coverage (UHC) and overarching aims of inclusivity and equity articulated across the Sustainable Development Goals.^7^,^9^ It is within this context that a focus on disability has been highlighted as a key consideration in the design, implementation and evaluation of programmes targeting neglected tropical diseases (NTDs).^9 10^ Given the disproportionate burden of both disability and NTDs among the poorest and most vulnerable members of society,^11 12^ the coverage of NTD control programmes and access to healthcare by people with disabilities have both been proposed as relevant ways in which UHC could be assessed and contribute towards scale-up.^13 14^

The barriers faced by people with disabilities in low-income and middle-income settings (LMIC) in accessing healthcare comprise a range of both demand and supply-side factors, including practical (eg, distance to facility), structural (eg, financial cost) and attitudinal (eg, discrimination).^15 16^ There is also growing evidence that people with disabilities are failing to be included fully in specific facility-delivered health services in LMIC such as sexual and reproductive health services or tuberculosis screening and treatment;^17 18^ in the implementation of water and sanitation programmes^19 20^ and exclusion resulting from the intersection of disability and gender.^21^ In contrast, relatively little research has investigated the accessibility of health services delivered by community health workers to people with disabilities^22^ or accessibility for children with disabilities.^23^ This evidence gap in part reflects assumptions that community health services are implicitly equitable by the nature of their design or delivery method.^24^,^26^ However, in the example of routine NTD control strategies such as mass drug administration (MDA), recent analysis has demonstrated important inequities that include considerable variation in coverage by wealth and gender.^27 28^

With substantial progress made towards universal primary education among LMICs over the past two decades, schools present a pragmatic and efficient platform for the delivery of basic health services.^29^ Control programmes targeting NTDs such as soil-transmitted helminths (STH) now routinely use school-based MDA as a mechanism to reach school-age (5–17 years) children (SAC).^30^ STH is among the most common parasitic infections globally, and SAC is prioritised in global policy recommendation as a high-risk group for STH-associated morbidity, which includes stunting, wasting and anaemia.^31^ While strategies such as school-based MDA do often attempt to address the reality that some children (including those with disabilities) will likely be excluded if they do not attend school—for example, by inviting non-attending children into school during deworming days or by including a community ‘mop-up’ component targeting out-of-school children—school-based deworming (SBD) is still likely to systematically exclude children with disabilities to some extent.^32^ Community-based MDA that is either delivered from a central point in a community or to households ‘door to door’ (as used by global strategies for lymphatic filariasis and onchocerciasis) potentially presents a more effective strategy for ensuring children with disabilities receive treatment for STH by overcoming the barriers that prevent children with disabilities from participating in education in the first place.^33^ However, if such strategies do not also sufficiently address these existing barriers experienced by people with disabilities when accessing healthcare—or introduce new barriers—such strategies are ultimately likely to undermine the effectiveness of NTD control programmes and present further obstacles to achieving UHC.

Here, we describe a nested study conducted within the Deworm3 Malawi trial, an ongoing randomised controlled trial with aims to assess the feasibility of interrupting STH transmission through biannual community-based MDA compared with routine, annual school-based MDA.^34^ The aim of this study was to quantify the prevalence of disability among children aged 5–17 years within the trial site, explore demographic factors associated with disability and assess the relative effectiveness of both school and community-based deworming (CBD) at reaching children with disabilities.

## Methods

### Ethical considerations

The parent trial of this study is registered at ClinicalTrials.gov (NCT03014167). This trial was approved by the College of Medicine Research Ethics Committee at the University of Malawi (P.04/17/2161), the London School of Hygiene and Tropical Medicine Observational/Interventions Research Ethics Committee (12013) and the Human Subjects Division at the University of Washington (STUDY00000180). A competent adult member of each household is required to provide written informed consent on behalf of the household when first enrolled in the community census of the trial and is then required to verbally reconfirm this consent at any related follow-up visits. A full description of how written informed consent is conducted for this study has previously been described.^35^

### Study setting and population

This study took place within the Malawi site of the Deworm3 trial, located within the Namwera health zone in Mangochi district, a rural area of southern Malawi.^34 36^ A baseline community census conducted between October and December 2017 enrolled a total of 131 074 individuals residing across 29 719 households in 124 villages.^35^ There are 49 public primary schools inside the study site or within 5 km of the study site boundary, in addition to six private primary schools and eight secondary schools. In line with national trends, reported enrolment in education in this census was consistently high (age-specific enrolment rate: 8 years (94%), 11 years (96%) and 14 years (89%)). Following a parasitological survey conducted between April and June 2018 which reported a baseline STH prevalence of 7.8% and hookworm as the predominant species of STH,^35^ the study site was subdivided into 40 clusters and randomly allocated 1:1 to either national standard of care MDA (annual SBD only) or community-wide MDA (annual SBD *plus* biannual CBD).

### Community census update design

All households within the study site were surveyed as part of a community census update conducted between April and June 2019. At each household identified, a trained enumerator accompanied by a village volunteer administered a modular census questionnaire using the SurveyCTO platform (Dobility). In households previously surveyed at baseline census, the census update consisted of confirming the residential status of each member, recording any additional household members, recollecting household-level reported access to water, sanitation and hygiene facilities; and observations of the materials used for construction of the dwelling. Households which had not been surveyed during baseline census completed a full census, which consisted of all elements described above in addition to a broader household-level questionnaire (eg, asset and livestock ownership, source of livelihood) and provision of a study identification card with a unique household number.^35^

### Child functioning survey module

As part of the community census update, all children aged 5–17 years enumerated within the study clusters were also surveyed using the Washington Group/UNICEF Child Functioning Module (CFM).^37 38^ This is a validated tool that has been designed to be used within population-based surveys to assess functional limitation in children, based on the conceptualisation of disability outlined in the International Classification of Functioning (ICF) developed by WHO.^39^ The CFM consists of 24 questions that assess 16 functional domains (eg, vision, hearing and mobility) selected from the ICF. Each domain is generally assessed in the form of a question related to perceived difficulty (eg, “*Does (name) have difficulty seeing*”) and a scaled series of responses (“*No difficulty*”, “*Some difficulty*”, “*A lot of difficulty*” and “*Cannot do at all*”). Where relevant, the domain is assessed with respect to any corrective aid (eg, “*When using their hearing aid,* d*oes (name) have difficulty hearing*”). The CFM was translated, pretested and delivered in line with interviewer guidelines^40^ .

### School-based deworming (routine standard of care)

Annual SBD is the predominant strategy globally for the routine delivery of preventive chemotherapy (PCT) for STH and schistosomiasis to SAC.^31 41^ Implementation of annual SBD in Malawi is delivered nationally by the National Schistosomiasis Control Programme (NSCP) with national coverage of 75% of SAC in 2017.^42^ At each school, implementation of SBD is led by a community health worker known as a *health surveillance assistant* (HSA) with the support of trained teachers. SBD takes place over 1 week, with 3 days of school-based treatment followed by 2 days of community ‘mop-up’ targeting children who are not enrolled or were absent. Treatment is with albendazole and praziquantel, with receipt of treatment recorded at the individual level in a school treatment register. For this study, the implementation of SBD was coordinated by the Blantyre Institute for Community Outreach (BICO) on behalf of the NSCP, although SBD was implemented according to routine national guidelines. With respect to the design of the parent trial, SBD was conducted in November 2019 across all study clusters.

### Community-based deworming (parent trial intervention)

Biannual CBD is a novel approach for the delivery of PCT for STH with albendazole to all eligible individuals over the age of 1 year, currently being evaluated as part of the Deworm3 trial.^34^ Implementation of CBD is at the village level and is led by the responsible HSA alongside a village volunteer and study officer. All eligible individuals in each household are offered treatment with albendazole only. The treatment is directly observed by the HSA and recorded for each individual by a trained study officer using an electronic treatment register developed for the trial.^43^ For the purpose of the Deworm3 trial, CBD was implemented by BICO who provide administrative support and substantial supervision. CBD was implemented the week immediately following SBD. With respect to the design of the parent trial, the analyses presented in this paper explore treatment coverage during the third (of a total of six biannual rounds of CBD) implemented for the trial in June/July 2019 in the 20 clusters randomised to intervention (annual SBD *plus* biannual CBD).

### Survey design

Prior to SBD taking place, children with disabilities (n=1467) and a random sample of children without disabilities (n=3769) who had reported attending a primary school during the census update had this confirmed at a follow-up school visit in September 2019. At each school, a roll call of all children was conducted class-by-class, followed by an inspection of enrolment registers, to verify whether they were enrolled or not. For those identified as no longer enrolled, the destination school (if known) was recorded. Children who were reported *not* to attend primary school during the census update were also followed up through a household visit to confirm whether they had since begun attending school. Where possible, a second set of school visits was conducted to confirm the enrolment of any child who had reported non-enrolment in the census but reported enrolment at the follow-up household visit, and a second set of household visits was conducted to confirm the status of any child who was not traced at the school reported in the census.

Treatments during SBD were assessed using two methods. First, direct observation of SBD was conducted by a study fieldworker placed in every school and central ‘mop-up’ distribution point. As each child was being treated, the study fieldworker cross-checked their reported name against a combined list of children with and without disabilities and subsequently recorded whether they were treated (or not) based on direct observation. Second, following the completion of SBD, any child that had not been directly observed during SBD was followed up for reported treatment by a subsequent survey visit within 1 week of SBD being completed at school (for those confirmed or reported to attend a school) or at the household (for those confirmed or reported not to attend a school, or who could not be traced at the school).

Owing to the high levels of treatment coverage, treatment during CBD was assessed using direct observation only, which was extracted from the electronic household treatment register completed as part of the broader trial. This register documents all eligible individuals as treated, not treated (eg, absent or refused), not eligible or followed up (ie, not visited or traced during CBD). For both SBD and CBD, study fieldworkers and those delivering treatment (ie, HSAs and teachers) were not aware of whether the child being observed had been defined as with or without disabilities during the child functioning survey, or as to the specific research objectives of this study.

### Analysis

We define disability as any surveyed child who was reported to have ‘a lot of difficulty’ or ‘cannot do at all’ in any domain of the CFM. Coverage of SBD was defined in two ways: (1) the number of children observed and identified divided by the total number of children in either study group and (2) the number of children who reported being treated during the follow-up survey over the number of children who were interviewed. Coverage of CBD was defined as the proportion of children in either study group who were recorded as treated in the individual treatment register divided by the total number of children in that study group. Ownership of household assets was used to construct a wealth index for each household using principal component analysis as previously described.^35^

Univariable associations between disability and individual and household-level characteristics were estimated using logistic regression. A priori interactions between age and sex were investigated, considering age as both a continuous variable, and by categorising into the three demographic groups used for stratification. We performed bivariate analyses (z-score) of the absolute proportion of children with and without disabilities treated during MDA (absolute differences in treatment) and logistic regression to examine whether disability status was associated with the likelihood of treatment during MDA (relative differences in treatment) and adjust for age, sex and reported school enrolment. Data management and analyses were performed using Stata V.17.0 (StataCorp, 2021; College Station, Texas, USA).

### Patient and public involvement

This study took place under the auspices of the Deworm3 Malawi Community Advisory Board (CAB), whose membership oversees all research activities conducted within the trial site. Members of the CAB were not involved in the development of the research question or choice of outcome measures specific to this study, but were closely engaged in the planning and implementation of the study.

## Results

### Baseline characteristics

A total of 44 574 children aged 5–17 years were surveyed for disability within the community census update. Overall 1467 (3.3%) children were identified as disabled in at least one domain (table 1). A greater proportion of boys had a disability (n=805, 3.6%) compared with girls (n=662, 3.0%), and the proportion of boys was higher in the majority (9/13) of domains. Among those with disabilities, the most frequently identified domains were remembering (n=336), hearing (n=334) and communication (n=282). Two-thirds of children with disabilities had limitations in one domain only (n=1012, 64.5%), and most children with disabilities had limitations in three or less domains (n=1390, 83.4%).

**Table 1:** Responses to CFM overall and disaggregated by sex

	Total	Male	Female
	(n=44 574)*	(n=22 468)	(n=22 103)
	N= (%)		
**Any domain:**	1467 (3.29)	805 (3.58)	662 (3.00)
Seeing	194 (0.44)	96 (0.43)	98 (0.44)
Hearing	334 (0.75)	180 (0.80)	154 (0.70)
Mobility	229 (0.51)	111 (0.49)	118 (0.53)
Self-care	169 (0.38)	90 (0.40)	79 (0.36)
Communication	282 (0.63)	160 (0.71)	122 (0.55)
Learning	263 (0.59)	143 (0.64)	120 (0.54)
Remembering	336 (0.75)	174 (0.77)	162 (0.73)
Attention and concentrating	174 (0.39)	79 (0.35)	95 (0.43)
Accepting change	212 (0.48)	105 (0.47)	107 (0.48)
Controlling behaviour	247 (0.55)	156 (0.69)	91 (0.41)
Making friends	174 (0.39)	99 (0.44)	75 (0.34)
Anxiety	256 (0.57)	131 (0.58)	125 (0.57)
Depression	195 (0.44)	101 (0.45)	94 (0.43)

* Sex unknown (n=3).

CFMChild Functioning Module

Children with disabilities were less likely to be female (adjusted OR (aOR)=0.83, 95% CI 0.75 to 0.93), and the prevalence of disability increased with age group (test for trend, p=0.03) (table 2). There was no evidence of an effect of age by sex for boys or girls, but girls were consistently less likely to be disabled than boys at 9–12 (aOR=0.79, 0.67–0.93) years and 13–17 (aOR=0.83, 0.68–0.99) years. While reported levels of school enrolment were generally high, there was a strong and consistent association between children with disabilities and school enrolment, with the likelihood of enrolment lower in each age group (eg, enrolment at age 8 years aOR=0.55, 0.34–0.87) compared with children without disabilities. Of those who were not currently enrolled, children with disabilities were less likely to have incomplete primary education (aOR=0.42, 0.31–0.56) or complete primary education (aOR=0.1, 0.01–0.75) compared with children without disability. The likelihood of disability was greater among children living in a rural area (aOR=1.36, 1.11–1.67) compared with an urban area, and there was weak evidence of a relationship between disability and increasing socioeconomic status, with the likelihood of disability greater among children in the least poor quintile (aOR=1.19, 1.01–1.4).

**Table 2:** Individual and household-level characteristics of all survey participants aged 5–17 years disaggregated by disability status and predictors of disability

		All children surveyed	Children with disabilities	Children without disabilities	Adjusted OR(95% CI)*	P value
		n=44 574	n=1467	n=43 107		
Sex:						
Male		22 468 (50.4)	805 (54.9)	21 663 (50.3)	1	–
Female		22 103 (45.6)	662 (45.1)	21 441 (49.7)	0.83 (0.75 to 0.93)	0.001
Age group:						
5–8 years		12 451 (27.9)	412 (28.1)	12 039 (27.9)	1	–
9–12 years		18 171 (40.8)	608 (41.5)	17 563 (40.7)	1.30 (1.05 to 1.60)	–
13–17 years		13 952 (31.3)	447 (30.5)	13 505 (31.3)	1.36 (0.93 to 1.99)	0.03
Effect of age by sex:						
Male	5–8 years	6268 (27.9)	219 (27.2)	6049 (27.9)	1	–
	9–12 years	9020 (40.2)	337 (41.9)	8683 (40.1)	1.20 (0.94 to 1.54)	0.15
	13–17 years	7180 (32.0)	249 (30.9)	6931 (32.0)	1.29 (0.86 to 1.95)	0.22
Female	5–8 years	6183 (28.0)	193 (29.2)	5990 (27.9)	1	–
	9–12 years	9148 (41.4)	271 (40.9)	8877 (41.1)	1.38 (1.09 to 1.76)	0.008
	13–17 years	6772 (30.6)	198 (29.9)	6574 (30.7)	1.42 (0.95 to 2.13)	0.09
Effect of sex by age:						
5–8 years	Male	6268 (50.3)	219 (53.2)	6049 (50.3)	1	–
	Female	6183 (49.7)	193 (46.8)	5990 (49.8)	0.91 (0.75 to 1.11)	0.35
9–12 years†	Male	9020 (49.6)	337 (55.4)	8683 (49.4)	1	–
	Female	9148 (50.3)	271 (44.6)	8877 (50.5)	0.79 (0.67 to 0.93)	0.005
13–17 years	Male	7180 (51.5)	249 (55.7)	6931 (51.3)	1	–
	Female	6772 (48.5)	198 (44.3)	6574 (48.7)	0.83 (0.68 to 0.99)	0.05
School enrolment:						
At age 5 years		4181 (57.0)	138 (50.7)	4043 (57.2)	0.76 (0.54 to 1.07)	0.12
At age 8 years		3995 (91.1)	150 (85.3)	3845 (91.3)	0.55 (0.34 to 0.87)	0.01
At age 11 years		3755 (91.6)	123 (80.5)	3632 (91.9)	0.36 (0.23 to 0.57)	<0.001
At age 14 years		3227 (85.9)	103 (76.7)	3124 (86.2)	0.52 (0.33 to 0.83)	0.006
Highest level of education (if not enrolled):						
None		4965 (62.9)	271 (74.7)	4694 (62.3)	1	–
Primary incomplete		2820 (35.7)	91 (25.1)	2729 (36.2)	0.42 (0.31 to 0.57)	–
Primary complete or higher		111 (1.4)	1 (0.3)	110 (1.5)	0.10 (0.14 to 0.76)	<0.001
Place of residence:						
Urban		4409 (9.9)	102 (7.0)	4307 (10.0)	1	–
Periurban		6073 (13.6)	277 (18.9)	5796 (13.5)	2.0 (1.59 to 2.51)	–
Rural		34 092 (76.5)	1088 (74.2)	33 004 (76.6)	1.36 (1.11 to 1.67)	<0.001
Socioeconomic status:						
Q1 (poorest)		8916 (20.0)	277 (18.9)	8639 (20.0)	1	–
Q2		8458 (18.9)	266 (18.1)	8192 (19.0)	1.03 (0.87 to 1.23)	–
Q3		9339 (21.0)	302 (20.6)	9037 (21.0)	1.08 (0.91 to 1.27)	–
Q4		8442 (18.9)	298 (20.3)	8144 (18.9)	1.20 (1.02 to 1.41)	–
Q5 (least poor)		9412 (21.1)	323 (22.0)	9089 (21.1)	1.19 (1.01 to 1.40)	0.11

* Adjusted for age, sex and school enrolment. All variables have complete data except for sex (three observations missing).

† Defined as a response of ‘a lot of difficulty’ or ‘cannot do’ in at least one domain of the CFM.

CFMChild Functioning Module

### Follow-up of children during SBD and CBD

**Figure 1:**
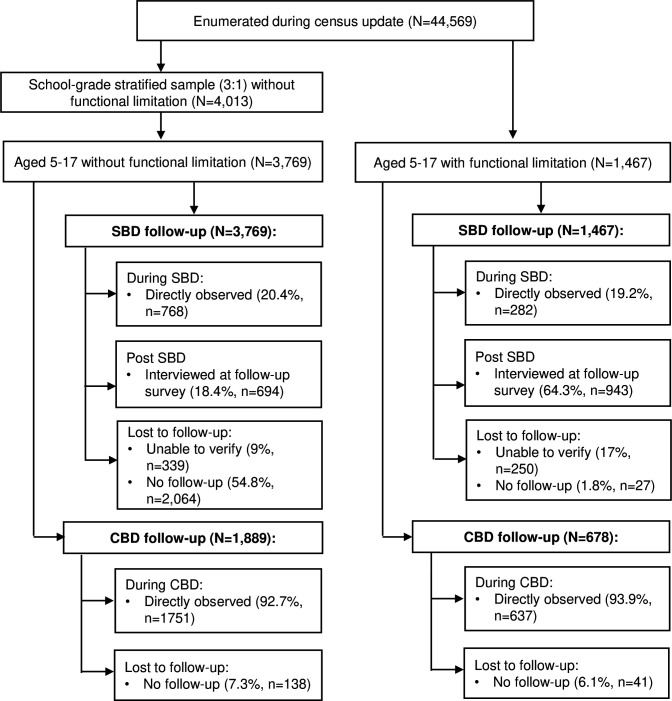
Study participant flow. CBD, community-based deworming; SBD, school-based deworming.

Overall, a lower-than-anticipated proportion of children was directly observed during SBD (figure 1). This proportion was similar but lower for children with disabilities (n=282, 19.2%) compared with children without disabilities (n=768, 20.4%). Most children with disabilities not identified during SBD were interviewed at a follow-up survey, with 943 (64.3%) interviewed at home or school, and a further 250 (17%) of children with disability traced but not found (eg, repeatedly absent at the time of household visit), and only 27 (1.8%) not traced. However, more than half of children without disabilities who were not identified during SBD were not traced at the follow-up survey (n=2064, 54.8%), with only 694 (18.4%) interviewed and a further 9% (n=339) traced but not interviewed. During direct observation of CBD, most children with disabilities (93.9%, n=637) and children without disabilities (92.7%, n=1889) were observed and identified; there were no significant differences in the characteristics of children observed compared with those not followed up.

### Absolute differences in treatment of children with disabilities during MDA

When assessed by direct observation of SBD, there was no significant difference in the absolute proportion of children with disabilities treated during SBD (n=277, 18.9%) compared with children without disabilities (n=750, 19.9%) with a total absolute difference of −1% (z-score=0.832, p=0.41) (table 3). After a follow-up of those not directly observed during SBD for reported treatment outcome, the proportion of children with disabilities reporting treatment (n=529, 56.1%) was significantly lower than the proportion of children without disabilities (n=458, 66.0%) with a total absolute difference of −9.9% (z-score=4.044, p<0.001). This difference was greater among girls (absolute difference=−11.9%, z-score=3.356, p<0.001) than boys (absolute difference=−8.2, z-score=2.420, p=0.02). The absolute proportion of all children treated during CBD was substantially higher than SBD. However, there was no significant difference in the absolute proportion of children with disabilities (n=613, 96.2%) treated compared with children without disabilities (n=1693, 96.7%) with an absolute difference of −0.5% (z-score=0.540, p=0.589).

**Table 3:** Absolute effect of disability on being treated through (1) school-based deworming and (2) community-based deworming, disaggregated by sex

	Children without disability	Children with disability	Difference(%)	z-score	P value
	n/N (%)	n/N (%)			
Treated at SBD (directly observed only)	750/3769 (19.9)	277/1467 (18.9)	−1.0	0.832	0.41
Male	355/1921 (18.5)	131/805 (16.3)	−2.2	1.373	0.17
Female	395/1848 (21.4)	146/662 (22.1)	−0.7	−0.365	0.71
Treated at SBD (follow-up survey of those not directly observed)	458/694 (66.0)	529/943 (56.1)	−9.9	4.044	<0.001
Male	225/345 (65.2)	304/533 (57.0)	−8.2	2.420	0.02
Female	233/349 (66.8)	225/410 (54.9)	−11.9	3.356	<0.001
Treated at CBD	1693/1751 (96.7)	613/637 (96.2)	−0.5	0.540	0.589
Male	847/882 (96.0)	325/338 (96.2)	+0.2	−0.098	0.92
Female	846/869 (97.4)	288/299 (96.3)	−1.1	0.916	0.36

CBDcommunity-based dewormingSBDschool-based deworming

### Relative differences in treatment of children with disabilities during MDA

There was no evidence of a difference in the likelihood of children with disabilities being treated during SBD when assessed by direct observation (unadjusted risk ratio (RR)=0.95, 95% CI 0.84 to 1.07) compared with children without disabilities (table 4). This result remained consistent after adjusting for age, sex and school enrolment (aRR=1.07, 95% CI 0.89 to 1.29). In contrast, there was a marginal difference in the likelihood of children with disabilities being treated during SBD when assessed by the report (follow-up of those not directly observed) (unadjusted RR=0.85, 95% CI 0.79 to 0.92), although after adjusting for covariates there was no further evidence of this association (aRR=0.95, 95% CI 0.80 to 1.12). There was no significant difference in the likelihood of children with disabilities being treated during CBD (unadjusted RR=1.01, 95% CI 0.98 to 1.04), which remained consistent in the adjusted analysis.

**Table 4:** Relative effect of disability on being treated through (1) school-based deworming and (ii) community-based deworming

	Model 1RR (95% CI)*	Model 2RR (95% CI)†	Model 3RR (95% CI)‡
Treated at SBD (directly observed only)			
Children without disabilities (n=3771)	1	1	1
Children with disabilities (n=1467)	0.95 (0.84 to 1.07)	1.02 (0.85 to 1.21)	1.07 (0.89 to 1.28)
Treated at SBD (follow-up survey of those not directly observed)			
Children without disabilities (n=694)	1	1	1
Children with disabilities (n=943)	0.85 (0.79 to 0.92)	0.82 (0.69 to 0.96)	0.95 (0.80 to 1.12)
Treated at CBD			
Children without disabilities (n=1889)	1	1	1
Children with disabilities (n=678)	1.01 (0.98 to 1.04)	1.04 (0.99 to 1.10)	1.04 (0.99 to 1.10)

All variables have complete data except sex (3three observations missing).

* Unadjusted.

† Adjusted for age and sex.

‡ Adjusted for age, sex and school enrolment.

CBDcommunity-based dewormingSBDschool-based deworming

## Discussion

This study of deworming of SAC in Mangochi district in southern Malawi found that despite consistently lower levels of school enrolment, there was no difference in the overall coverage or likelihood of treatment for children with disabilities compared with children without disabilities within both routine school-based and novel community-based MDA. We observed no significant difference in the proportions of children with disabilities treated during SBD compared with children without disabilities when assessed by direct observation and identification, although coverage for both groups was relatively low. While coverage of children with disabilities was higher when assessed by recall of SBD, after controlling for relevant covariates this result was consistent with the primary outcome of direct observation and identification. Absolute coverage of all children was substantially higher during trial-delivered community-based MDA with no difference between those with and without disabilities. This study contributes to the limited and mixed body of evidence on the relative coverage of children with disabilities in LMIC by healthcare services,^44^ and is one of very few to assess the coverage of healthcare services either among SAC or of non-facility-(ie, community or school)based services. These results also contribute to a better understanding of how recent policy calls to more fully consider^45^ and systematically report^46^ the health outcomes of children with disabilities can be achieved.

Our study confirmed the strong and consistent association seen globally between disability and worse educational engagement including enrolment and participation.^5 47^ Age-specific enrolment of children with disabilities was lower at all age points. Additionally, we observed that enrolment began to decrease from 11 years for children with disabilities compared with 14 years for children without disabilities. This potentially presents a risk to school health programmes that specifically target older children such as SRH, compounding inequities that are faced by people with disabilities in accessing these services.^18^ Despite low enrolment rates, however, we did not see lower levels of coverage of children with disabilities by a campaign-based (ie, short duration) school deworming programme. Nevertheless, low enrolment rates likely still present a major risk to the effectiveness of health programmes that rely on sustained long-term coverage such as school feeding.^48^

Within studies relating to coverage of community-level health services, a multicountry study that incorporated data from 2005 found children with disabilities aged 2–4 years were less likely to have received vitamin A supplementation in five of the 10 countries assessed.^49^ More recently, however, two studies assessing childhood vaccination among children with disabilities in Cameroon^50^ and Kenya^51^ found no difference in the proportions treated. Among coverage of other healthcare services, the results of this study broadly align with research from a variety of settings that found children with disabilities were no less likely to have sought healthcare when sick,^47 51 52^ despite facing a range of additional barriers.^15 16^ While the study populations are not directly comparable to this study, research among children with disabilities with specific classifications of disability has found lower coverage among children with intellectual disabilities who sought care for fever,^53^ but was the same among children with hearing impairments who had received testing for HIV.^54^ In addition, multiple studies of dental care among children with intellectual or behavioural disabilities have described settings of both lower coverage^55 56^ and equal coverage^57 58^ of routine care.

While this study found that children with disabilities were no less likely to have been treated during MDA, this study did not explore broader issues of healthcare access beyond coverage, including utilisation and quality. Evidence demonstrates that children with disabilities may have additional health needs, such as an increased risk of undernutrition^59^ or recent serious illness,^47^ and concomitantly a greater utilisation of health services, including the risk of hospitalisation.^60 61^ As such, this study did not assess differences in the health status or needs of children with disabilities, including the presence of STH infection or related health behaviours. While the role of sanitation in the transmission of STH is well understood, our research in this study site has previously found that this was not a risk factor for STH infection and described a setting of relatively high coverage levels of basic sanitation at the household level.^35^ However, as evidence suggests that people with disabilities may face poorer quality of access within their household, in addition to the specific challenges that may be faced by people with disabilities in accessing sanitation in Malawi,^20 62^ this remains an important aspect in assessing whether the two deworming strategies assessed are equitable for children with disabilities.

A major strength of this study is that it was conducted as part of a thorough census of a community of 130 000 individuals and included a rigorous assessment of disability by way of the CFM tool. Developed in 2016 in response to a lack of standardisation in the assessment of disability globally,^63^ this deployment of the CFM is substantially larger than use in any previous epidemiological survey^64^,^66^ and comparable in scale to a nationally representative survey,^67^ demonstrating the feasibility and utility of the CFM as part of a comprehensive evaluation of a public health intervention. The CFM was adapted from previous work in developing a disability question set based on the ICF for use with adults (Washington Group Short Set on Functioning) and underwent extensive validation and testing prior to release.^68 69^ The use of a mother or primary caregiver as the respondent has subsequently been demonstrated to be reliable and consistent with teacher or child (self) reporting,^70 71^ and the CFM is consistent with routine tools used to assess specific aspects of disability among pre-SAC.^66 72^ As the control (standard-of-care) arm of the broader trial, school-based MDA was implemented in routine practice without any additional input from the study team, permitting reasonable comparison of the results of this study with the programme as implemented nationally. Finally, by leveraging the randomised design and data collection methods of the parent trial, our study also uses a ‘gold standard’ outcome of direct observation to estimate the treatment of children within community-based MDA, in contrast to self-reported or proxy measures for treatment.^73^

This study has a number of limitations, including the relatively low proportion of children directly observed during school-based MDA compared with previous estimates,^74^ suggesting that a substantial proportion of children were not identified during their treatment, leading to our use of a less accurate self-reported treatment as an additional measure of school-based MDA. Our experience in Malawi of attempting to identify children at school by using their names as reported at their households is that this has many practical challenges related to individual preferences and naming practices.^75^ Reliable estimates of coverage of school-based MDA have previously been identified as a major programmatic issue,^76^ and as such feasible measures of estimating the coverage of SBD beyond reported treatment at postcoverage surveys remain challenging. In addition, this study did not specifically consider the additional benefit of the community mop-up component of school-based MDA in reaching children with disabilities specifically given their consistently lower participation in schooling, although given high levels of absenteeism more generally^77^ this element is likely to remain a key component of school-based MDA in Malawi. Finally, as community-based MDA was conducted in the context of a large cluster-randomised trial evaluating this strategy, the high coverage achieved among both children with and without disabilities cannot necessarily be extrapolated to other community-based health services (eg, distribution of long-lasting insecticidal nets) delivered by HSAs as part of their routine activities.

In conclusion, this study found no difference in the proportion of children with disabilities treated during routine school-based MDA, despite demonstrating consistently lower levels of participation in education, and that community-based MDA when delivered by a trial with consistently high population coverage did ensure equitable treatment of all children regardless of disability. Disability in childhood can feasibly be assessed as part of large-scale population surveys, and provides a useful indicator of intervention equity given the multiple disadvantages faced by children with disabilities. However, we suggest that disability should be more closely considered in the design, implementation and monitoring of school-health interventions given this consistent difference in educational participation, which may result in more pronounced differences where the intervention requires sustained attendance (eg, school feeding) or where coverage levels are generally higher (eg, health education as part of the routine curriculum).

## Data Availability

Data are available upon reasonable request.
