# Supplementary material for: Disability in childhood and the equity of health services: a cross-sectional comparison of mass drug administration strategies for soil-transmitted helminths in southern Malawi
Source: BMJ Open. 2024 Sep 5;14(9):e083321. doi: 10.1136/bmjopen-2023-083321 (PMC11381638; doi:10.1136/bmjopen-2023-083321)
Supplement: Supplementary file 1 [file bmjopen-2023-083321.reviewer_comments.pdf]

PEER REVIEW HISTORY

BMJ Open publishes all reviews undertaken for accepted manuscripts. Reviewers are asked to complete a checklist review form (**http://bmjopen.bmj.com/site/about/resources/checklist.pdf**) and are provided with free text boxes to elaborate on their assessment. These free text comments are reproduced below.

ARTICLE DETAILS

**TITLE (PROVISIONAL)**	Disability in childhood and the equity of health services: a cross-sectional comparison of mass drug administration strategies for soil-transmitted helminths in southern Malawi
**AUTHORS**	Witek-McManus, Stefan; Simwanza, James; Msiska, Rejoice; Mangawah, Hastings; Oswald, William; Timothy, Joseph; Galagan, Sean; Shaikh, Mariyam; Pearman, Emily; Legge, Hugo; Walson, Judd; Juziwelo, Lazarus; Davey, Calum; Pullan, Rachel; Bailey, Robin; Kalua, Khumbo; Kuper, Hannah

VERSION 1 – REVIEW

**REVIEWER**	Simkiss, Doug<br>University of Warwick, Warwick Medical School
**REVIEW RETURNED**	19-Apr-2024

**GENERAL COMMENTS**	This was an interesting study on a topic that warrants highlighting much more; health equity in general and the issues of equity for children with disability in particular.<br><br>The authors may be interested in a new framework for the social determinants of health (in a UK setting) published by Barnardo's and the Institute for Health Equity in February this year and I am attaching two reports from that team.<br><br>My chief concern with the paper probably relates to the timing of this paper in relation to the main paper from the trial. As the latter does not seem to be published yet, there is necessarily quite a lot of detail in this paper on the broader context, that should be in the main trial paper. Is that paper due for publication soon and, if so, could it be cited to reduce the detail in this paper?

**REVIEWER**	Al-Delaimy, Ahmed K<br>Anbar University
**REVIEW RETURNED**	29-Apr-2024

**GENERAL COMMENTS**	Thank you for writing about this field of research.<br>I have some comments:<br>- The title is a mixed two subjects, I think it would be more scientific attractive title to include one main subject. The present one is a mixer of health services, disability and deworming for STHs diseases. To have a precise title it is much better to concentrate on one subject.

	- School health services or finding disable school child in a research study it is better to be isolated from deworming subject! Because each of them is a big issue.<br>- Deworming subject should have a t- test significant to find results before and after deworming for the same child and this study was done in Malaysia in 2013 you can get back to it and I will put the reference below.<br>- Although the author mentioned that they follow the Washington group. UNICEF Module on child functioning, but any handicapped children (somatic, neurological) children were missed! And what about stuttering and speech disorder of children?<br>I suggest the author should mention the criteria of chosen the present disabilities in the manuscript and the reason of not mentioning other disabilities studied in other research papers.<br>- Tables are busy and need to be minimize in numbers<br>- Some reference ae missed and it is important in such topic for example:<br>- nothing mentioned about the effect of STHs on child education in school<br>1- Epidemiology of intestinal polyparasitism among Orang Asli school children in rural Malaysia<br>2- Developing and Evaluating Health Education Learning Package (HELP) to Control Soil- Transmitted Helminthiasis among Orang Asli Children in Malaysia<br>3- https://pubmed.ncbi.nlm.nih.gov/34860479/

VERSION 1 – AUTHOR RESPONSE

Response to reviewer 1:

This was an interesting study on a topic that warrants highlighting much more; health equity in general and the issues of equity for children with disability in particular. The authors may be interested in a new framework for the social determinants of health (in a UK setting) published by Barnardo's and the Institute for Health Equity in February this year and I am attaching two reports from that team.

We thank the reviewer for sharing this reference. We have reflected on the policy implications of this within the discussion section of this manuscript. We have also been made aware of consideration of disability in the recently published Global Action for Measurement of Adolescent health (GAMA) which we have incorporated into our discussion and link here for the reviewers knowledge - https://www.who.int/groups/the-global-action-for-measurement-of-adolescent-health

My chief concern with the paper probably relates to the timing of this paper in relation to the main paper from the trial. As the latter does not seem to be published yet, there is necessarily quite a lot of detail in this paper on the broader context, that should be in the main trial paper. Is that paper due for publication soon and, if so, could it be cited to reduce the detail in this paper?

A manuscript describing the primary outcome of the parent trial (change in STH prevalence between study arms) is currently in preparation, however, this only provides a very brief description of the two interventions as implemented generally (i.e. across all three study sites) rather than how they were implemented in the Malawi site specifically, which we think is necessary context for interpretation of the results presented. We also think that given the audience for this manuscript may not necessarily

be those working directly in STH control that a longer description of the interventions would be useful. There is minimal discussion of STH in the introduction or discussion sections (e.g. epidemiology or pathology of STH infection). We have however reviewed the sections of the manuscript that overlap with the parent trial (3.1-3.3, 3.5-3.6) and edited these as much as possible to be more concise.

Reviewer: 2

- The title is a mixed two subjects, I think it would be more scientific attractive title to include one main subject. The present one is a mixer of health services, disability and deworming for STHs diseases. To have a precise title it is much better to concentrate on one subject.
- School health services or finding disable school child in a research study it is better to be isolated from deworming subject! Because each of them is a big issue.

We thank the reviewer for these two comments and will consider them together in this reply. We agree that the title and body of this manuscript covers a number of areas, each of which would have merit as a research focus on its own. However, we think that the added value and strength of this manuscript is specifically that we have brought these intersecting characteristics together into one cohesive analysis that has relevance to a much broader audience. We agree that the equity of school health services in aspects other than disability should be the focus of further research, but that is beyond the scope of the survey dataset presented here. While presenting only the demographics and factors associated with disability amongst children would also be of relevance by itself, we think it is important to present this alongside results that demonstrate what this actually means in the context of routine health service delivery. In this way these results have relevance beyond both disability studies and STH or deworming, given the scope of community and school-based approaches in many health systems, but also for those with a broader interest in the equity of health systems, given discussion for disability as a proxy for equity of health systems (references 13 and 14).

- Deworming subject should have a t- test significant to find results before and after deworming for the same child and this study was done in Malaysia in 2013 you can get back to it and I will put the reference below.

This study was not intended to assess STH infection, or the treatment efficacy of albendazole, amongst children with disabilities. The baseline parasitological survey of the study site conducted prior to implementation of community-based deworming (https://doi.org/10.1371/journal.pntd.0009292) did not collect information on disability status and so we are unfortunately unable to explore this relationship further. The main trial design (repeat cross-sectional) also precludes individual-level assessment of infection status before and after the intervention. Given the significance of relative poverty as both a major risk factor for STH infection and strongly associated with disability status, we recognise why the reviewer has raised this question and hope that this manuscript will encourage future school-based STH prevalence surveys - which remain the main monitoring tool for STH control programmes - to include assessment of disability amongst children. This point is also raised in the discussion, paragraph 4.

- Although the author mentioned that they follow the Washington group. UNICEF Module on child functioning, but any handicapped children (somatic, neurological) children were missed! And what about stuttering and speech disorder of children? I suggest the author should mention the criteria of chosen the present disabilities in the manuscript and the reason of not mentioning other disabilities studied in other research papers.

We did not exclude children on the basis of any specific clinical conditional in this study, and all children were eligible for the survey. The Washington Group/UNICEF Child Functioning Module (CFM) uses a framework based on the International Classification of Functioning (ICF), Disability and Health, which conceptualises disability (referred to as functioning) as an interaction between an individual's health condition, environmental factors, and personal factors. This is in contrast to a specifically medical or social model of disability. To take the example given of a speech disorder, this would likely be captured across several of the ICF domains including communication ("When (name) speaks, does he/she have difficulty being understood by people…") but also for example learning ("Compared with children of the same age, does (name) have difficulty learning things?") if for example, the individual did not consider the speech disorder to prevent them from communicating, but did prevent them from sufficiently participating in a classroom lesson. It is not impossible that some children with disabilities on the basis of a given clinical definition may have been missed by the CFM, but we think this is likely to be a very small number and ultimately would not undermine the results we present, which aim to look at the relationship between disability and health and education outcomes in an unbiased way.

- Tables are busy and need to be minimize in numbers.

We have reviewed the tables and maintain that all four tables are relevant to the main body of the manuscript, however, we have made a number of minor edits to the tables for clarity and hope this will make them easier for a reader to take in.

- Some reference ae missed and it is important in such topic for example:
- nothing mentioned about the effect of STHs on child education in school
1- Epidemiology of intestinal polyparasitism among Orang Asli school children in rural Malaysia
2- Developing and Evaluating Health Education Learning Package (HELP) to Control Soil-Transmitted Helminthiasis among Orang Asli Children in Malaysia
3- https://pubmed.ncbi.nlm.nih.gov/34860479/

This comment is well noted. We have revised the introduction section to briefly describe better context to STH, including key references to the health consequences of STH infection, and the methods sections to include the epidemiology of STH in this specific study setting.

Editors comments:
• Please ensure that your abstract is formatted according to our Instructions for Authors: http://bmjopen.bmj.com/pages/authors/#research. This has been done, please see lines 37-67 in the track-change manuscript.

• Please add a section entitled 'Strengths and limitations of this study' (immediately after the abstract). This section should contain up to five short bullet points, no longer than one sentence each, that relate specifically to the methods. The novelty, aims, results or expected impact of the study should not be summarised here. This has been done, please see lines 68-80 in the track-change manuscript.

• Please remove the "What is already known on this topic/What this study adds/How this study might affect research, practice or policy" sections as these are not part of journal formatting requirements. This has been done, please see lines 81-101 in the track-change manuscript.

• Please ensure that you have fully discussed the methodological limitations of the study in the Discussion section of the main text. This has been better signposted at the beginning of the relevant paragraph, please see lines 449-451 in the track-change manuscript.

VERSION 2 – REVIEW

**REVIEWER**	Simkiss, Doug<br>University of Warwick, Warwick Medical School
**REVIEW RETURNED**	27-Jun-2024

**GENERAL COMMENTS**	The authors have addressed the issues raised by the reviewers and the editorial team and I think this paper should now be accepted for publication.

**REVIEWER**	Al-Delaimy, Ahmed K<br>Anbar University
**REVIEW RETURNED**	14-Jun-2024

**GENERAL COMMENTS**	I think the author should change the title of the manuscript. In the objectives of the study the author mentioned the first objective is prevalence of disabilities and the associated factors? and then mentioning the 2nd objective which is comparison between the School and Community deworming program among disabled children. I did not observed details about the prevalence and associated factors mentioned in the objective of the study? to my opinion the author mixed two different items together which is: prevalence and mass drug comparison between two deworming programs.<br>So I suggest to remove any thing related to prevalence and associated factors, because this is another study and starting from the title to the references, the reviewer and reader cannot find highlighted figures or tables and or discussion related to this topic.<br>Also the author should mentioned clearly and specifically the test and table related to comparison between the two programs.<br>Some important refences are missing related directly to the topic done in villages of Malaysia for example:<br>https://www.ncbi.nlm.nih.gov/pmc/articles/PMC4261692/<br>https://pubmed.ncbi.nlm.nih.gov/25144662/

VERSION 2 – AUTHOR RESPONSE

Response to reviewer 2:

I think the author should change the title of the manuscript. In the objectives of the study the author mentioned the first objective is prevalence of disabilities and the associated factors? and then mentioning the 2nd objective which is comparison between the School and Community deworming program among disabled children. I did not observed details about the prevalence and associated factors mentioned in the objective of the study? to my opinion the author mixed two different items

together which is: prevalence and mass drug comparison between two deworming programs. So I suggest to remove any thing related to prevalence and associated factors, because this is another study and starting from the title to the references, the reviewer and reader cannot find highlighted figures or tables and or discussion related to this topic.

Table 1 is a descriptive table of the cohort of children with disabilities and prevalence of each domain (i.e. the specific functional limitation). Table 2 then describes the cohort of children with disabilities overall and factors associated with disability. These two tables are described in the text under results section 4.1. (baseline characteristics) with reference to the relevant table. We have reviewed the tables and text and believe that this is clear. As written in our previous response, we think this information about children with disabilities is both useful and necessary for proper interpretation of the results subsequently presented in table 3 and 4 (objective 2) which present the relative coverage of children with disabilities compared to children without disabilities by type of deworming programme.

Also the author should mentioned clearly and specifically the test and table related to comparison between the two programs. Some important refences are missing related directly to the topic done in villages of Malaysia for example: https://www.ncbi.nlm.nih.gov/pmc/articles/PMC4261692/

https://pubmed.ncbi.nlm.nih.gov/25144662/

We refer the reviewer to section 3.8 (analysis) where the analysis approach used to compare the different deworming strategies are described. We have also reviewed the section headings and table titles, and believe these clearly describe the results being presented. The suggested references are interesting but are not relevant to this manuscript. The first relates to programme effectiveness of an STH programme with an outcome of STH infection (rather than coverage of said programme by specific demographic characteristic(s) as was the focus of this study). The second suggested reference is the specific epidemiology of STH in a setting which is not comparable to Malawi; we have cited the appropriate comparable paper (reference 35) in the text to give context to the reader of the epidemiology of STH in this setting.
